# Parental Corporal Punishment and Peer Victimization in Middle Childhood: A Sex-Moderated Mediation Model of Aggression

**DOI:** 10.3389/fpsyg.2020.573329

**Published:** 2021-02-26

**Authors:** Alba Martin, José Manuel Muñoz, Paloma Braza, Rosa Ruiz-Ortiz, Nora del Puerto-Golzarri, Eider Pascual-Sagastizábal, Aitziber Azurmendi, Rosario Carreras

**Affiliations:** ^1^Department of Psychology, University of Cádiz, Cádiz, Spain; ^2^Department of Basic Psychological Processes and Their Development, Faculty of Psychology, University of the Basque Country, Donostia-San Sebastián, Spain

**Keywords:** corporal punishment, physical aggression, relational aggression, peer victimization, moderated mediation analyses, middle childhood, parenting

## Abstract

There is a peak in peer victimization during middle childhood, with multiple negative consequences. Parental use of corporal punishment and child aggression are the most widely studied predictors of this phenomenon. The aim of the present study was to analyze whether parental use of corporal punishment affects peer victimization through child aggression. This mediation model was explored for both mothers and fathers and for both physical and relational forms of aggression and peer victimization. Furthermore, we also analyzed whether the mediation models were moderated by the sex of the child. Participants were 234 third graders (46% girls). Child aggression and victimization were measured by peers using the Mini Direct Indirect Aggression Inventory. Independent measures of mother’s and father’s use of corporal punishment were obtained from a PCA of items from the Parental Styles and Dimensions Questionnaire (PSDQ). Conditional process modeling was carried out using a macro for SPSS developed by Hayes (2013). Results indicated that aggression mediated the relation of parental corporal punishment to peer victimization. Some interesting moderating effects of sex in this mediation model were found; specifically, physical, and relational aggression mediated the relation of maternal corporal punishment to peer victimization only in boys. Few studies to date have addressed the connection between aggressive behavior and peer victimization as outcomes of corporal punishment, taking into consideration the role of parent’s and child’s sex, and both physical and relational forms of aggression and victimization during childhood.

## Introduction

Peer victimization refers to a situation in which someone is the target of frequent aggressive behaviors by peers (Crick et al., 2007). In contrast to bullying victimization, peer victimization does not imply a power imbalance between perpetrator and victim and is more commonly reported (Söderberg and Björkqvist, 2019). Victimization experiences have been found to reach a peak in middle childhood (Sumter et al., 2012), and research has shown that, during this developmental stage, up to 60% of children are exposed to some form of victimization (Kochenderfer-Ladd and Wardrop, 2001). The negative effects of peer victimization on psychological well-being and adjustment have been widely documented (Hawker and Boulton, 2000; Cook et al., 2010; Kochel et al., 2012; Rudolph et al., 2014; Holt, 2015), and these adverse outcomes, such as low self-esteem, peer rejection, anxiety, and depression, can persist over time (Ttofi et al., 2011; Hymel and Swearer, 2015). Being victimized by peers is especially hurtful during adolescence (Salmivalli, 2018), and longitudinal research has shown that peer victimization tends to remain stable from middle childhood onward, a period during which peer relations become increasingly important (Sourander et al., 2000). Thus, if we want to prevent the serious consequences of this phenomenon, it is important to study the factors that may place children at risk for peer victimization in middle childhood.

Corporal punishment, considered a parental disciplinary strategy, is still frequently used today, and research suggests that a high percentage of children are physically punished by their parents during childhood (Gershoff, 2010; Wilson et al., 2018). However, there is a growing consensus regarding the links between corporal punishment and increased risk for detrimental child outcomes (Gershoff and Grogan-Kaylor, 2016). Some empirical works have addressed peer victimization as an outcome of corporal punishment (Schwartz et al., 1997; Duong et al., 2009; Lereya et al., 2013; Söderberg et al., 2016), but it remains unclear why corporal punishment appears in some cases to be an antecedent of victimization and what the mediating mechanisms are that explain this relationship (Khademi et al., 2018). Given that the relationship between corporal punishment and aggressive behavior is well-documented (Gershoff, 2002; Lansford et al., 2011; MacKenzie et al., 2015), and the role of aggression as an antecedent of victimization has been highlighted by numerous empirical works (Kochenderfer-Ladd and Wardrop, 2001; Yeung and Leadbeater, 2007), the aim of this study was to examine the mediating role of peer aggression in the association between corporal punishment and peer victimization.

Moreover, the present study also aims to fill some other important gaps in the literature. First, the majority of early research into the effects of corporal punishment on maladjustment has focused on mothers’ use of this practice, and much less is known about the impact of fathers’ use of this disciplinary strategy (Lee et al., 2015). Second, despite theoretical and empirical support for sex differences in aggression, victimization, and corporal punishment (Chung et al., 2009; Ostrov and Godleski, 2010; Xing and Wang, 2013), the findings of studies examining the role of sex in the effects of corporal punishment on peer aggression or victimization have been limited and mixed (Del Hoyo-Bilbao et al., 2018; Manring et al., 2018). Finally, little is known about the association between relational child aggression and corporal punishment, as well as about how this type of aggression affects the impact of this negative parenting strategy on maladjustment (Micalizzi et al., 2017). In contrast to physical aggression, in which damage is physical, relational aggression includes behaviors intended to damage victims through the peer group, such as spreading rumors or encouraging others to avoid them. However, some authors have suggested that physical aggression and relational aggression follow different trajectories across development (Dodge et al., 2006; Murray-Close et al., 2007), and others have reported sex-linked differences (Archer, 2004) and differential associations with maladjustment (Card et al., 2008).

Thus, the present study also examined whether the relationship between maternal and paternal use of corporal punishment and peer victimization, mediated by child aggression, is moderated by the child’s sex. Moreover, both physical and relational forms of aggression and victimization were included.

### Parental Use of Corporal Punishment and Child Aggression

Corporal punishment is a parenting strategy which includes spanking, slapping, smacking, and grabbing a child (Orue et al., 2016). The acceptability and use of this particular form of discipline have begun to decline over recent years due to appeals by many researchers, practitioners and human rights organizations, and a growing number of countries have prohibited its use through policies and laws (Österman et al., 2014; Lansford, 2019). However, even in some countries in which this practice has been legally banned, many children continue to experience corporal punishment (Lansford et al., 2017). In relation to the use of corporal punishment by parents, fathers have been less studied than mothers (e.g., Berlin et al., 2009; Gershoff et al., 2012, Gromoske et al., 2012; Lee et al., 2013), and the results of research including both parents have been inconsistent. While some studies suggest that mothers use corporal punishment more frequently than fathers (Wolfner and Gelles, 1993; Hallers-Haalboom et al., 2016), others report nonsignificant differences in the frequency of corporal punishment use by mothers and fathers (Wissow, 2001; Mehlhausen-Hassoen, 2019). Regarding the sex of the child, most authors have found that boys suffer corporal punishment more frequently and more severely than girls during middle childhood, although the effect sizes reported are generally small (Millichamp et al., 2006; Lansford et al., 2010, Lansford, 2019). Gershoff (2002) argues that boys tend to engage more in externalizing behaviors than girls and that these behaviors elicit corporal punishment.

More than 50 years’ worth of research attests to the detrimental effects of corporal punishment on children (Vaughan-Eden et al., 2019). While most previous studies fail to consider the severity of the punishment (Gershoff, 2013), recent evidence suggests that the negative outcomes associated with mild or severe physical punishment are actually quite similar (Gershoff and Grogan-Kaylor, 2016; King et al., 2018). For example, corporal punishment has been considered an important predictor of child aggression, with spanking children at age 1 year being related to externalizing behavior one and 2 years later (Berlin et al., 2009; Gromoske et al., 2012). Moreover, Thompson et al. (2017) found that preschoolers whose parents used corporal punishment were more aggressive than other children. In a meta-analysis that tested age as a moderator of the association between corporal punishment and children’s externalizing behavior, the association was found to be stronger when children were 10–12 years of age than when they were either younger or older (Gershoff, 2002). Corporal punishment has been hypothesized to predict increases in child aggression because it models aggression (e.g., Bandura and Walters, 1973); is related to social information processing deficits which in turn predict aggressive behavior (Weiss et al., 1992); and initiates coercive cycles of aversive behaviors between parent and child (Patterson et al., 1992).

It is still unclear how parents’ and children’s sex interacts with corporal punishment to predict child aggression. For example, Deater-Deckard and Dodge (1997) found that the association between corporal punishment and aggression was stronger when the parent and child were the same sex. McKee et al. (2007) observed that both fathers’ and mothers’ physical discipline was similarly related to daughters’ and sons’ externalizing problems. MacKenzie et al. (2012) found that mothers’ spanking was more strongly related to children’s aggression than fathers’ spanking. Given these mixed findings, the present study also aims to help clarify the relationship between corporal punishment and child aggression in mother–son, mother–daughter, father–son, and father–daughter dyads.

Finally, although the association between negative parenting and children’s physical aggression has been well-documented, the influence of adverse parenting on the development of relational aggression has been less studied (Kawabata and Crick, 2016). Some researchers have analyzed the association between physical punishment and a global measurement including both physical and relational aggression (Söderberg et al., 2016), and several studies have found that parenting characterized by psychological control increases children’s relational aggression (Kawabata et al., 2011; Kuppens et al., 2013). However, the literature review revealed that only a few authors have analyzed the association between corporal punishment and relational aggression. For example, Zhu et al. (2019) found that corporal punishment had an impact on both physical and relational aggression among children. Furthermore, although some authors have argued that relational aggression is more typical among girls than among boys (Kistner et al., 2010), the few studies examining whether pathways from harsh discipline to relational aggression differ by sex have reported mixed findings. Thus, Nelson and Crick (2002) found that mothers’ use of corporal punishment was only positively associated with relational aggression among boys, whereas Spieker et al. (2012) found that early harsh maternal control predicted relational aggression only for girls. Finally, Zulauf et al. (2018) found that corporal punishment directly predicted relational aggression regardless of the child’s sex. Further research is therefore required to clarify the effects of corporal punishment on relational aggression, taking into account the sex of both parent and child.

### Child Aggression and Peer Victimization

Despite the well-established relationship between child aggression and peer victimization, contradictory conclusions have been drawn regarding the direction of the effects involved (Cooley et al., 2018). Peer socialization theory suggests that students who are victimized may learn from these interactions and model the aggressive behaviors in future hostile interactions with peers (Rose and Rudolph, 2006). Thus, in accordance with this theory, some researchers have found that peer victimization leads to aggressive behavior (Barker et al., 2008). However, other researchers view aggression as an antecedent of victimization (Salmivalli and Helteenvuori, 2007; You and Yoon, 2016). Thus, previous longitudinal studies have shown a link between aggressive behavior and prospective levels of peer victimization in early and middle childhood (Snyder et al., 2003; Hanish et al., 2004; van Lier and Koot, 2010), with no significant sex differences being found in the association between aggression and victimization (Ostrov and Godleski, 2013; Cooley et al., 2018; Lam et al., 2018).

Moreover, this association between aggression and victimization may depend on the type of behavior in question, physical, or relational (Lam et al., 2018). According to the specificity hypothesis of aggression (Crick et al., 1999; Sullivan et al., 2006; Ostrov, 2008), each form of aggression is positively associated only with the same form of victimization. Our study therefore analyzes both physical and relational aggression and victimization separately.

### Child Aggression as a Mediator Between Corporal Punishment and Peer Victimization

Several empirical studies have shown that corporal punishment by parents predicts peer victimization. For example, Schwartz et al. (1997) reported that peer-victimized children suffered more punitive experiences at the hands of their parents than other children; Duong et al. (2009) found that maternal corporal punishment was a risk factor for peer victimization among adolescents; Björkqvist and Österman (2014) reported higher rates of physical punishment among victims of school bullying than among those not exposed to bullying; and Barker et al. (2008) used a longitudinal design to show that high levels of harsh reactive parenting predicted peer victimization in preschool.

Different mediators have been proposed to explain the mechanism that underlies the relationship between parental use of corporal punishment and victimization by peers, including depression (Yabko et al., 2008; Hong et al., 2012) and psychosomatic symptoms (Lamela and Figueiredo, 2013). A recent avenue of research views child aggression as a mediator in the association between corporal punishment and victimization. Anthonysamy and Zimmer-Gembeck (2007) found that maltreatment by parents was indirectly related to children’s peer rejection through children’s physical and verbal aggression, and Söderberg et al. (2016) found that the effect of physical punishment on victimization was partially mediated by aggressive behavior. Comparatively, less is known about the role of children’s relational aggression as an explanatory factor for the association between harsh parenting and peer relationships (Wang, 2017). Further research is required to test the pathway from corporal punishment to peer victimization via child aggression. The present study aims to help clarify this question by including relational (as well as physical) forms of aggression and victimization, with the sex of both parent and child as moderators.

### The Present Study

**FIGURE 1:**
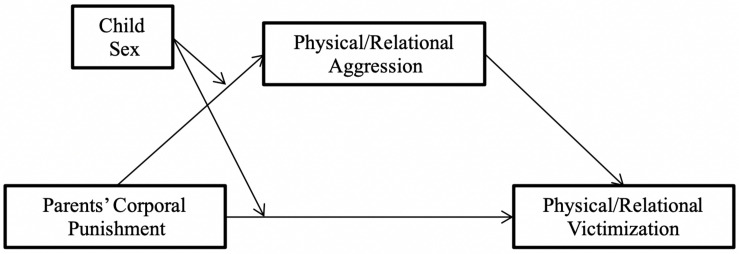
The summarized conceptual model of moderated mediation.

The aims of this study were (1) to explore the mediating role of child aggression in the relationship between parents’ use of corporal punishment and peer victimization and (2) to explore the moderating role of the sex of the child in this mediation. These analyses were carried out for aggression and peer victimization, both physical and relational, and for fathers and mothers. Four conditional models (Figure 1) were proposed for the study: (1) a moderated mediation model of father’s corporal punishment (henceforth FCP), physical aggression and physical victimization (henceforth PA and PV, respectively), and child’s sex; (2) a moderated mediation model of FCP, relational aggression and relational victimization (henceforth RA and RV, respectively), and child’s sex; and (3) and (4), which are identical to models (1) and (2) (respectively), but with mother’s corporal punishment (henceforth MCP) as a predictor instead of FCP.

## Materials and Methods

### Participants and Procedure

Participants were 279 children, recruited from twelve third-grade classrooms in eight public elementary schools in two Spanish cities. All schools in these cities were asked to participate and eight finally accepted. Parents and teachers were informed of the research aims in a meeting. Anonymity and confidentiality procedures were carefully explained in the written consent form approved by the Commission on Ethics in Research and Teaching at the University of the Basque Country. Ten children were absent during the administration of the questionnaire, and 35 parents did not return the questionnaires. The final sample comprised 234 children, evenly divided in terms of sex (46% girls) and with a mean age of 8.15 years (*SD* = 1.23). Of the final sample, 18% were only children, while 12% had more than two siblings. The majority of parents (66%) had post-school qualifications.

At the beginning of the academic year, fathers and mothers received a personalized version of the Parental Styles and Dimensions Questionnaire (PSDQ; Robinson et al., 2001) and were asked to complete it separately. During the last month of the academic year, children of each classroom collectively completed the Mini Direct Indirect Aggression Inventory (Mini-DIA; Österman and Björkqvist, 2008) guided by two researchers; they were asked about the frequency with which each of their peers exhibited the aggressive or victimization behaviors.

### Measures

#### Family Demographic Survey

Parents completed a short sociodemographic survey designed to gather information about the age of the children, number of siblings, and parents’ education level.

#### Parental Use of Corporal Punishment

Corporal punishment was assessed on the basis of the responses given by fathers and mothers (separately) to the items on the Parental Styles and Dimensions Questionnaire (PSDQ; Robinson et al., 2001). This questionnaire includes 62 items, rated on a Likert-type scale ranging from 1 “never” to 4 “always,” designed to determine how often parents exhibit certain behaviors toward their children. Recent studies have found high levels of reliability and validity for this questionnaire (Olivari et al., 2013; Lee and Brown, 2018).

A Principal Component Analysis using a varimax (orthogonal) rotation was carried out, revealing a four-factor solution for the PSDQ completed by fathers, with a good matrix indicator (K-M-O = 0.86; Bartlett sphericity x2 [df = 190] = 1259.25, *p* < 0.001) accounting for 50.64% of the total variance. The same procedure was repeated for mothers’ data, obtaining a three-factor solution with good matrices indicator (K–M–O = 0.85; Bartlett sphericity x2 [df = 231] = 1319, 83, *p* < 0.001), accounting for 42.44% of the total variance. The factors Father’s corporal punishment (FCP) and Mother’s corporal punishment (MCP) included items reflecting parent’s use of corporal punishment with their children (e.g., I spank when our child is disobedient).

#### Physical and Relational Aggression and Victimization

These two forms of aggression and victimization were measured using the Mini Direct Indirect Aggression Inventory (Mini-DIA; Österman and Björkqvist, 2008). The Mini-DIA is an abbreviated version of the Direct-Indirect Aggression Scales (Björkqvist et al., 1992), developed as a less time-consuming version of the original instrument. This peer estimation inventory comprises 6 items rated on a Likert-type scale (ranging from 0 “Never” to 4 “Very often”) which ask respondents about certain behaviors demonstrated by their peers in relation to aggression and victimization. Items related to physical aggression (“Has he/she hit, kicked, or shoved someone?”), relational aggression (“Has he/she gossiped maliciously about someone, spread harmful rumors about someone, or tried to socially exclude someone?”), physical victimization (“Someone has ever hit, kicked or pushed him/her?”), and relational victimization (“Someone has gossiped maliciously about him/her, spread harmful rumors about him/her or tried to socially exclude from others?”) were used. We found data to be reliable for this sample (Cronbach’s alpha was 0.96 for aggression and 0.83 for victimization), and the validity of inventory has been evidenced by the fact that it yields similar results as the original DIAS (Österman, 2010).

### Data Analysis

All statistical procedures were conducted using IBM SPSS Statistics 25. Descriptive statistics, sex differences (identified using one-way ANOVAs), and Pearson correlations were calculated for the study variables. The PROCESS macro for SPSS (Model 4; Hayes, 2013) was used to determine the mediation effects of child aggression on FCP/MCP-peer victimization relationships. The mediation analysis was performed for both forms (physical and relational) of aggression and victimization. Model 8 (Hayes, 2013) of the same macro was used to test the moderating effects of child’s sex in the tested mediation models. For these PROCESS models, a bootstrapping procedure was selected with 10000 bootstrap samples used to calculate bias-corrected 95% confidence intervals (CIs). A significant effect was considered to exist if the CIs did not include zero.

## Results

### Preliminary Analyses

Means, standard deviations, one-way ANOVA tests, and Pearson correlations for the study variables are reported in Table 1. Boys scored significantly higher than girls for PA, RA, PV, and RV. Boys also scored higher than girls for FCP, although no differences were found between the sexes for MCP. As expected, MCP correlated with PA, RA, PV, and RV; however, FCP was associated with PA, RA, and RV, but not with PV.

**TABLE 1:** Descriptive statistics, ANOVA tests, and Pearson correlations for the study variables.

	Boys M (SD)	Girls M (SD)	*F* test	1	2	3	4	5
1. FCP	0.203 (1.13)	−0.253(0.734)	12.601**	−				
2. MCP	0.050 (1.04)	−0.059(0.949)	0.726	0.405**	−			
3. PA	0.919 (0.754)	0.390 (0.450)	47.820**	0.180**	0.245**	−		
4. RA	0.647 (0.602)	0.471 (0.412)	13.210**	0.143*	0.261**	0.793**	−	
5. PV	0.979 (0.620)	0.546 (0.472)	41.460**	0.110	0.180**	0.785**	0.656**	−
6. RV	0.653 (0.566)	0.498 (0.401)	6.608**	0.152*	0.147*	0.605**	0.670**	0.665**

FCP, father’s corporal punishment; MCP, mother’s corporal punishment; PA, child’s physical aggression; RA, child’s relational aggression; PV, physical victimization; RV, relational victimization. *p < 0.05, **p < 0.01.

### Father’s Corporal Punishment and Peer Victimization Mediated by Child Aggression

**FIGURE 2:**
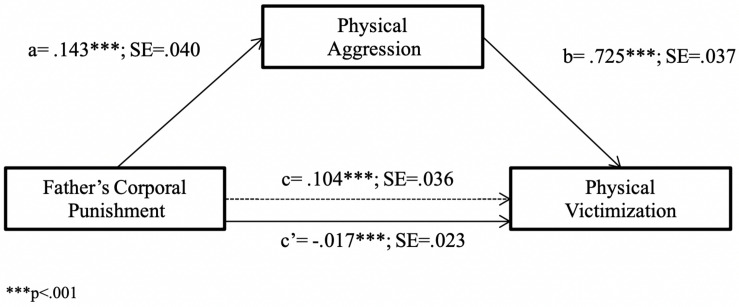
Physical victimization predicted from father’s corporal punishment through physical aggression. ***p < 0.001.

First, the hypothesis positing that child’s PA mediates the relationship between FCP and PV was tested using Model 4 of the PROCESS macro (Hayes, 2013). FCP was found to have an indirect effect on PV through its association with child’s PA [*R*^2^ = 0.64, *F*(2,231) = 203.96, *p* < 0.001). As depicted in Figure 2, the direct effect of FCP on PV was positive and significant (path c); FCP was also a positive and significant predictor of child’s PA (path a); when the effect of FCP was partialled out, child’s PA was found to positively and significantly predict PV (path b). Finally, when the effect of the mediator variable (child’s PA) was partialled out, the relationship between FCP and PV was no longer significant (path c′). Using bootstrapping, the indirect effect of FCP on PV through child’s PA was found to be significant [*B* = 0.10, SE = 0.03, 95% CI = (0.04, 0.17)], suggesting that the link between FCP and PV is mediated by child’s PA.

Second, the moderating role of child’s sex in the previous mediation model was explored using Model 8 of the PROCESS macro (Hayes, 2013). Specifically, we calculated the moderating effect of child’s sex on the relationship between FCP and child’s PA, as well as on the relationship between FCP and PV. The results revealed that the interaction between FCP and child’s sex did not significantly predict either child’s PA or PV (Table 2) and that the sex difference in the indirect effect of FCP on PV through child’s PA was not significant [index of moderated mediation = −0.07, SE = 0.05, 95% CI = (−0.18, 0.03)]. However, although the sex difference observed was not significant, the 95% bias-corrected bootstrap-CI indicated that the indirect effect of FCP on PV via child’s PA was stronger for boys than for girls. Thus, for boys, the indirect relationship between FCP and PV was significant [*B* = 0.08, SE = 0.04, 95% CI = (0.01, 0.17)], whereas for girls it was not [*B* = 0.01, SE = 0.04, 95% CI (−0.06, 0.08)].

**TABLE 2:** Moderated mediation models for father’s corporal punishment, physical aggression, physical peer victimization, and sex.

Predictor	Mediator physical aggression			Criterion physical victimization		
	B	SE	CI	B	SE	CI
FCP	0.068	0.044	[−0.019, −0.155]	–0.027	0.026	[−0.080, 0.024]
Child’s sex	–0.457	0.078	[−0.611, −0.303]	–0.051	0.050	[−0.149, 0.047]
FCP × child’s sex	–0.100	0.088	[−0.274, 0.074]	–0.027	0.053	[−0.131, 0.077]
*R*^2^		0.175			0.640	
*F*		16.309***			101.911***	

FCP, father’s corporal punishment. ***p < 0.001.

**FIGURE 3:**
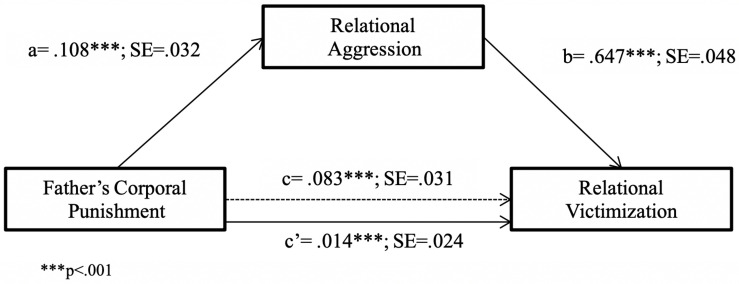
Relational victimization predicted from father’s corporal punishment through relational aggression. ^∗∗∗^*p* < 0.001.

Next, the same analyses (Models 4 and 8 of the PROCESS macro; Hayes, 2013) were carried out to test the mediation model of the impact of FCP on RV through child’s RA, and the moderating effect of child’s sex in this mediation model. As shown in Figure 3, FCP was found to have an indirect effect on child’s RV through its impact on child’s RA (*R*^2^ = 0.46, *F*(2,231) = 97.07, *p* < 0.001). Further, the bootstrapped 95% CI confirmed that this indirect effect was significant [*B* = 0.14, SE = 0.06, 95% CI = (0.03, 0.26)].

The results of Model 8 (Table 3) revealed that the product of FCP and child’s sex had no significant predictive effect on either child’s RA or RV [index of moderated mediation = −0.04, SE = 0.05, 95% CI (−0.14, 0.04)]. However, the indirect effect of FCP on RV through child’s RA was different for boys and girls, with the 95% bias-corrected bootstrap CI indicating that this indirect effect was only significant for boys (*B* = 0.07, SE = 0.04, 95% CI = [0.00, 0.15], for boys; *B* = 0.02, SE = 0.03, 95% CI = [−0.03, 0.08], for girls).

**TABLE 3:** Moderated mediation models for father’s corporal punishment, relational aggression, relational peer victimization, and sex.

Predictor	Mediator relational aggression			Criterion relational victimization		
	B	SE	CI	*B*	SE	CI
FCP	0.070	0.037	[−0.003, 0.143]	0.023	0.028	[−0.031, 0.078]
Child’s sex	–0.191	0.065	[−0.320, −0.062]	0.028	0.050	[−0.072, 0.123]
FCP × child’s sex	–0.067	0.074	[−0.217, 0.078]	0.028	0.055	[−0.081, 0.137]
*R*^2^		*0*.*082*			0.457	
*F*		6.878**			48.328***	

FCP, father’s corporal punishment. ***p < 0.001.

### Mother’s Corporal Punishment and Peer Victimization Mediated by Child Aggression

**FIGURE 4:**
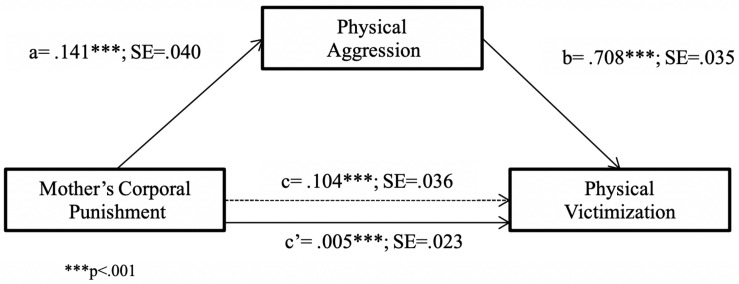
Physical victimization predicted from mother’s corporal punishment through physical aggression. ^∗∗∗^*p* < 0.001.

Similarly, models 4 and 8 of the PROCESS macro were used (respectively) to test the hypothesis positing that child’s aggression mediates between MCP and peer victimization and the one positing that this mediation model is moderated by the child’s sex, first in relation to physical aggression and victimization, and then in terms of their relational counterparts. MCP was found to have an indirect effect on PV through child’s PA (*R*^2^ = 0.64, *F*(2,239) = 208.89, *p* < 0.001). As shown in Figure 4, the direct effects of MCP on PV (path c) and of MCP on child’s PA (path a) were positive and significant. Furthermore, when the effect of MCP was partialled out, child’s PA was found to positively and significantly predict PV. Finally, when the effect of the mediating variable (child’s PA) was partialled out, the relationship between MCP and PV was no longer significant (path c′). Using bootstrapping, the indirect effect of MCP on PV through child’s PA was found to be significant [*B* = 0.10, SE = 0.03, 95% CI = (0.04, 0.16)], thus confirming that the association between MCP and PV is mediated by child’s PA.

**FIGURE 5:**
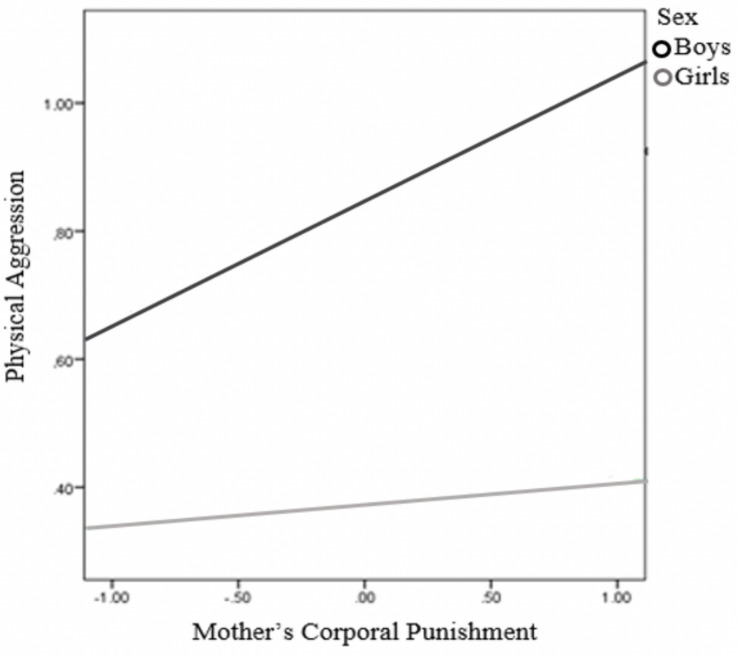
Child’s sex as a moderator between mother’s corporal punishment and physical aggression.

Model 8 of the PROCESS macro was used to test whether child’s sex moderated the previous mediation model. Specifically, we estimated the moderating effect of child’s sex on the relationship between MCP and child’s PA, as well as on the relationship between MCP and PV. The results revealed (Table 4) that the interaction between MCP and child’s sex significantly predicted child’s PA, but not PV. Moreover, the effect of MCP on child’s PA was significant only for boys (the conditional effects of the focal predictor at moderator values were *B* = 0.20, SE = 0.05 *p* < 0.001 for boys, and *B* = 0.03, SE = 0.058, *p* = .56 for girls). For descriptive purposes, we plotted PA against MCP separately for boys and girls (Figure 5). Furthermore, the difference between the indirect effects for boys and for girls was significant [index of moderated mediation = −0.11, SE = 0.05, 95% CI = (−0.21, −0.03)]. The 95% bias-corrected bootstrap-CI indicated that the indirect effect of MCP on PV through child’s PA was significant only for boys (*B* = 0.13, SE = 0.04, 95% CI = [0.06, 0.22], for boys; *B* = 0.02, SE = 0.02, 95% CI = [−0.01, 0.07], for girls).

**TABLE 4:** Moderated mediation models for mother’s corporal punishment, child’s physical aggression, physical peer victimization, and sex.

Predictor	Mediator physical aggression			Criterion physical victimization		
	*B*	SE	CI	*B*	SE	CI
MCP	0.114	0.038	[0.040, 0.190]	0.008	0.023	[−0.038, 0.053]
Child’s sex	–0.473	0.075	[−0.621, −0.324]	–0.088	0.048	[−0.183, 0.007]
MCP × child’s sex	–0.161	0.076	[−0.312,−0.011]	0.018	0.045	[−0.072, 0.108]
*R*^2^		0.195			19.273***	
*F*		0.641			106.030***	

MCP, mother’s corporal punishment. ***p < 0.001.

**FIGURE 6:**
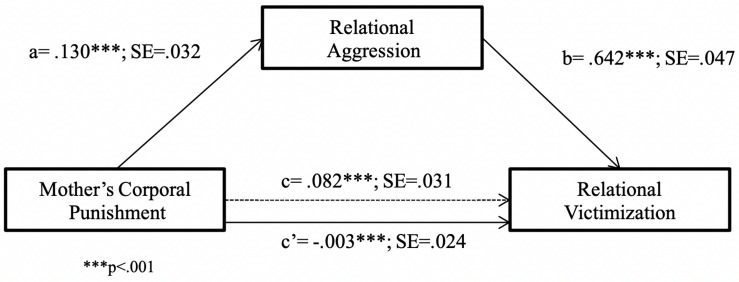
Relational victimization predicted from mother’s corporal punishment through relational aggression. ^∗∗∗^*p* < 0.001.

Lastly, as regards relational forms of aggression and victimization, the tested mediation model confirmed that MCP also exerted an indirect effect on RV through child’s RA [*R*^2^ = 0.45, *F*(2,239) = 99.33, *p* < 0.001]. Further, the bootstrapped 95% CI confirmed that this indirect effect was significant [*B* = 0.08, SE = 0.02, 95% CI = (0.04, 0.14)], as shown in Figure 6.

**FIGURE 7:**
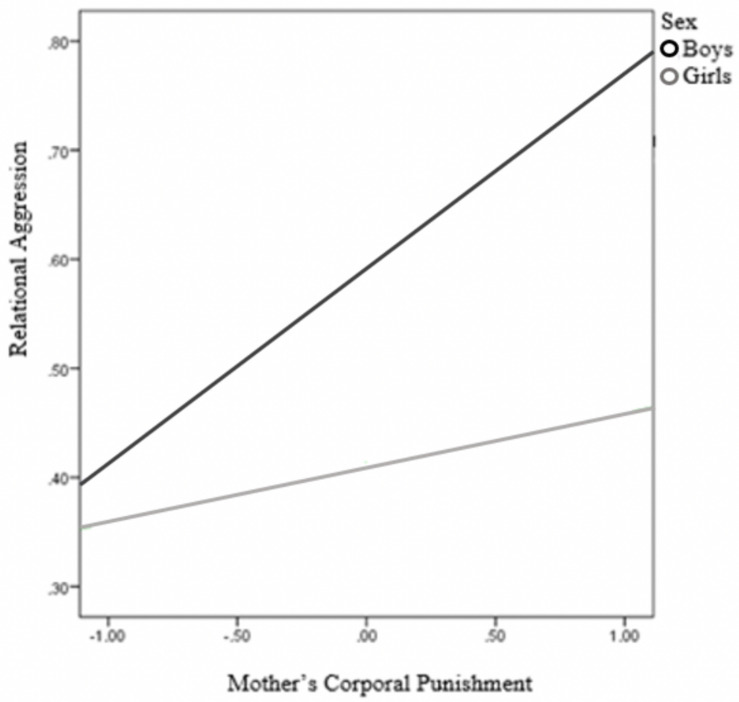
Child’s sex as a moderator between mother’s corporal punishment and relational aggression.

Again, the presence of mediated moderation was tested using the conditional process model (Model 8), analyzing the moderating effect of child’s sex on the relationship between MCP and child’s RA, as well as on the relationship between MCP and RV. The results revealed that child’s sex had an effect on the association between MCP and child’s RA (Table 5), with this effect being significant only for boys (the conditional effects of the focal predictor at moderator values were *B* = 0.18, SE = 0.04, *p* < 0.001 for boys, and *B* = 0.05, SE = 0.05, *p* = .310, for girls; RA against MCP is shown separately for boys and girls in Figure 7). Furthermore, the index of moderated mediation of child’s sex was significant [*B* = −0.08, SE = 0.03, 95% CI = (−0.16, −0.01)], indicating that the indirect effect of MCP on RV through child’s RA was significant only for boys (*B* = 0.16, SE = 0.03, 95% CI = [0.05, 0.19], for boys; *B* = 0.03, SE = 0.02, 95% CI = [−0.00, 0.07], for girls).

**TABLE 5:** Moderated mediation models for mother’s corporal punishment, child’s relational aggression, relational victimization, and sex.

Predictor	Mediator relational aggression			Criterion relational victimization		
	*B*	SE	CI	*B*	SE	CI
MCP	0.114	0.031	[0.052,−0.176]	–0.000	0.024	[0.048, −0.047]
Child’s sex	–0.183	0.062	[−0.307, −0.066]	–0.005	0.048	[0.099, −0.088]
MCP x Child’s sex	–0.128	0.063	[−0.253, −0.003]	0.024	0.048	[−0.070, −0.119]
*R*^2^		0.111			0.455	
*F*		9.948***			49.378***	

MCP, Mother’s corporal punishment. ***p < 0.001.

## Discussion

The aim of the present study was to analyze whether parental use of corporal punishment affects peer victimization through its impact on child aggression. As a further step, we also analyzed whether the sex of the child moderated the mediating effect of child aggression in the relation between corporal punishment and peer victimization. These models were tested for both mothers and fathers and for both physical and relational aggression and peer victimization. Our findings indicate that (1) the adverse effect of parental use of corporal punishment on peer victimization was explained by child aggression; and (2) the influence of mother’s corporal punishment on peer victimization through child aggression was moderated by the child’s sex, meaning that (a) associations between mother’s corporal punishment and both physical and relational aggression were only significant for boys and (b) the indirect effect of mother’s corporal punishment on physical/relational victimization through physical/relational aggression was significant, again, only for boys.

Our first finding suggests that child aggression can be considered an explanatory mechanism in the association between parental use of corporal punishment and peer victimization. In other words, parents who frequently use corporal punishment as a disciplinary strategy may exacerbate their children’s aggressive behavior, which in turn increases the risk of their being victimized by peers. It is interesting to note that these relationships supported a fully mediated model, meaning that most of the effect of corporal punishment on peer victimization occurs as a result of the increase in child aggression. Some researchers have reported a direct association between physical punishment and peer victimization, even after controlling for children’s externalizing behaviors, and they have speculated that children who are subjected to physical punishment may develop a kind of “victim personality” that frequently elicits victimization by others (Bowes et al., 2009; Lereya et al., 2013; Björkqvist and Österman, 2014). However, in these studies the authors have focused on harsher forms of corporal punishment, such as physical abuse or maltreatment and their associations with bullying victimization, a specific type of victimization that involves a power imbalance between the perpetrator and the victim. Since bullying victimization and peer victimization are partly overlapping but not identical constructs (Söderberg and Björkqvist, 2019), our findings highlight the importance of differentiating, in future research, between victimization due to peer aggression and victimization due to bullying, as well as between corporal punishment and other more severe forms of abuse, in order to gain a better understanding of the specific mechanisms that explain the relationships which exist between corporal punishment and victimization.

Moreover, our results support a mediation model for both physical and relational forms of aggression and victimization. Why would parents’ use of corporal punishment make children more aggressive in both physical and relational terms? It seems logical to assume that parental use of corporal punishment may prompt children to become more physically aggressive themselves, and indeed, this pathway has been well-documented (Coie and Dodge, 1998; Ladd and Pettit, 2002, for reviews). By comparison, less is known about the link between corporal punishment and relational aggression (Sandstrom, 2007), although this association has been reported by recent studies (Zulauf et al., 2018; Zhu et al., 2019). As Choe et al. (2013) suggest, parental physical discipline probably models both relational and physical aggression, since this type of discipline provides parents with a forceful power for obtaining immediate compliance and allows them to demonstrate their relational and physical dominance over children.

Furthermore, as regards the moderating role of child’s sex in maternal mediation models, our results reveal that the indirect effects of corporal punishment on peer victimization through child aggression were significant only for boys. Interestingly, the same result was found for both physical and relational forms of aggression and victimization. Although previous studies have reported more relational aggression among girls during adolescence (Dinić et al., 2014; Björkqvist, 2018), in middle childhood, boys are more physically and, furthermore, as or even more relationally aggressive as/than girls (Olweus and Kallestad, 2010). It seems that, during this developmental stage, the form of aggression/victimization (physical or relational) does not differentially explain the association between mother’s corporal punishment and peer victimization. Why then were the indirect effects of corporal punishment on peer victimization through child aggression significant for boys but not for girls? Some studies have found that the effect of corporal punishment on aggressive behavior is stronger for boys (Straus and Stewart, 1999; Xing et al., 2011; Velki, 2019), while the impact on internalizing behavior problems is stronger for girls (Sternberg et al., 2006; Xing and Wang, 2013). Moreover, research has consistently shown that many internalizing symptoms (e.g., anxiety or depression) are associated with victimization (Casper et al., 2017). Considering all this together, it seems that, among girls, internalizing rather than externalizing behaviors are more likely to explain the relationship between corporal punishment and victimization. Indeed, some researchers (Yabko et al., 2008) have found that the use of corporal punishment (power-assertive parenting) leads to internalizing problems (depression) which, in turn, contribute to victimization.

Finally, it is important to note that the mediation model linking father’s corporal punishment, child aggression, and peer victimization was not moderated by the sex of the child. Indeed, child’s sex did not moderate the direct association between father’s corporal punishment and child aggression. Corporal punishment is a disciplinary strategy that sons experience more frequently than daughters, particularly in the context of milder forms of physical punishment in middle childhood (Straus and Stewart, 1999; Bardi and Borgognini-Tarli, 2001). It is likely that, because boys generally behave more aggressively and are harder to discipline, both mothers and fathers tend to use harsher methods of parenting (including corporal punishment) more often with their sons than with their daughters (Maccoby and Jacklin, 1978; Parke and Slaby, 1983). Despite this, however, Mehlhausen-Hassoen (2019) suggested that fathers restrained themselves considerably more than mothers when dealing with their daughters. Together, these factors could explain why, in our study, child’s sex was not found to moderate the relationship between father’s corporal punishment and child aggression, prompting us to hypothesize that, in this association, child’s sex explains more than it moderates.

In spite of the findings discussed above, the study has several limitations that should be acknowledged. First, the data gathered were cross-sectional in nature, meaning that our conclusions should be interpreted with care. Exploring the associations observed in the present study using a longitudinal design would enable future research to further our understanding of the direction of the relationships which exist between corporal punishment, aggression and victimization, and its development over time. Second, the generalizability of our findings is also limited due to the characteristics of the study sample, which was community-based with low prevalence of aggression. One noteworthy strength of our study is that it uses peer estimations of aggression and victimization, rather than self-reports based on a single participant’s potentially biased view. Other strengths include the assessment of both physical and relational peer aggression; the participation of relatively equal numbers of boys and girls; and the fact that the sex of both parents and children was taken into account.

In sum, the study supports the idea that physical punishment is an inappropriate mean of parenting, since our results show that even milder forms of corporal punishment by parents impact peer victimization through child aggression (both physical and relational). These results highlight the importance of parental training in positive parenting strategies that inhibit the development of aggressive behaviors in children and protect them against the adverse effects of peer victimization. Moreover, our findings also provide evidence of the relevance of children’s and parents’ sex in the context of these relationships. Thus, in relation to mothers, the child’s sex affects the association in such a way that aggression explains the relationship between punishment and victimization only in boys. In relation to fathers, we believe that the sex of the child may explain more than moderate the association between corporal punishment and child aggression: fathers use corporal punishment with boys but not with girls, and being a boy is an important predictor of aggression. Future research should study the role of variables such as internalizing problems or warm parenting in the association between corporal punishment and peer victimization in girls, since, according to recent research, among girls, the impact of corporal punishment on internalizing behavior problems is stronger and lack of parental warmth may be a significant predictor of peer aggression.

## Data Availability Statement

The raw data supporting the conclusions of this article will be made available by the authors, without undue reservation.

## Ethics Statement

The studies involving human participants were reviewed and approved by Commission on Ethics in Research and Teaching at the University of the Basque Country (M10/2017/101). Written informed consent to participate in this study was provided by the participants’ legal guardian/next of kin.

## Author Contributions

AM conceived the presented idea and performed the computations. RC and JM supervised the development of the idea and hypothesis and the interpretation of the results. AM, RC, and JM wrote the manuscript. All authors discussed the results and contributed to the final manuscript.

## Conflict of Interest

The authors declare that the research was conducted in the absence of any commercial or financial relationships that could be construed as a potential conflict of interest.
